# Topology of WC/Co Interfaces in Cemented Carbides

**DOI:** 10.3390/ma16165560

**Published:** 2023-08-10

**Authors:** Boris B. Straumal, Lev N. Shchur, David G. Kagramanyan, Elizaveta P. Konstantinova, Alexander V. Druzhinin, Alexei N. Nekrasov

**Affiliations:** 1 Chernogolovka Scientific Center, Osipyan Institute of Solid State Physics, Russian Academy of Sciences, Ac. Osipyan Str. 2, 142432 Chernogolovka, Russia; lshchur@hse.ru (L.N.S.); dgkagramanyan@hse.ru (D.G.K.); epkonstantinova@hse.ru (E.P.K.); druzhinin@issp.ac.ru (A.V.D.); 2 Department of Applied Mathematics, HSE University, Myasnizkaja Str. 20, 101000 Moscow, Russia; 3 Korzhinsky Institute of Experimental Mineralogy, Russian Academy of Sciences, Ac. Osipyan Str. 4, 142432 Chernogolovka, Russia; alexei.nekrasov@iem.ac.ru

**Keywords:** cemented carbides, equilibrium shape, interphase boundaries, machine vision, cobalt matrix, tungsten carbide

## Abstract

WC–Co cemented carbides build one of the important classes of metal matrix composites. We show in this paper that the use of machine vision methods makes it possible to obtain sufficiently informative statistical data on the topology of the interfaces between tungsten carbide grains (WC) and a cobalt matrix (Co). For the first time, the outlines of the regions of the cobalt binder were chosen as a tool for describing the structure of cemented carbides. Numerical processing of micrographs of cross sections of three WC–Co alloys, which differ in the average grain size, was carried out. The distribution density of the angles in the contours of cobalt “lakes” is bimodal. The peaks close to 110° (so-called outcoming angles) correspond to the contacts between the cobalt binder and the WC/WC grain boundaries. The peaks close to 240° (or incoming angles) correspond to the WC “capes” contacting the cobalt “lakes” and are determined by the angles between facets of WC crystallites. The distribution density of the linear dimensions of the regions of the cobalt binder, approximated with ellipses, were also obtained. The distribution density exponentially decreases with the lengths of the semi-axes of the ellipsoid, approximating the area of the cobalt binder. The possible connection between the obtained data on the shape of cobalt areas and the crack trajectories in cemented carbides is discussed.

## 1. Introduction

WC–Co cemented carbides build one of the important classes of metal matrix composites. The first patent for cemented carbides based on tungsten carbide with a cobalt binder was registered 100 years ago, on 23 March 1923 in Germany [1]. The unique combination of strength (>3000 MPa at Co content of 10 wt.%) [2], high density (≈15 g/cm^3^ [2,3]), fracture toughness (>10 MPa·m^1/2^ depending on size of WC grains [4]), abrasion resistance and hardness (up to 20 GPa [2]) makes WC–Co cemented carbides irreplaceable for numerous applications [5,6]. 

The main structural element of cemented carbides are the WC grains (which are hard but brittle). In turn, they are surrounded by the ductile metallic binder phase. The binder is usually cobalt-based [7,8,9,10,11,12] but also can consist of Cr [10], Cu [11,13], Al [14,15], nickel or Ni-based alloys [12,16,17,18,19] or iron [16] or Fe-based alloys such as Fe–Ni, Fe–Ni–Co, Fe–Cr, Fe–Al [20], Fe–Ni–C [21], Fe–Ni, Fe–Ni–Co, Fe–Mn [17,22], Fe–Ni–Co [8,23], Fe–Cr–Ti(C,N) [23], Fe–Al–B [24], FeAl, Ni_3_Al [18], Fe–Ni–Cr [19,25], Fe–Ni–Co [9], Fe–Cu [26], FeAl [27,28], Fe–Mn [29,30], Fe–Ni–C [31], H13 Hudson tool steel [32], AISI 304 stainless steel [33,34] and high vanadium tool steels such as PM10V and PM 15 V [35]. 

**Figure 1:**
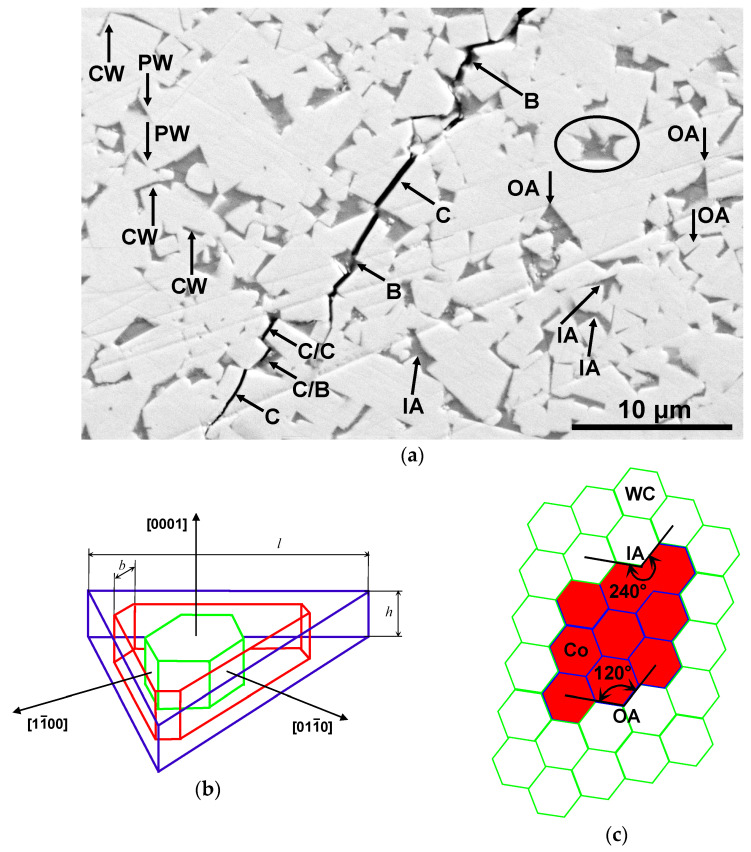
(**a**) SEM micrograph of a WC–Co alloy with various structural elements. Center: crack growth trajectory. The symbol C denotes a transgranular fracture; C/B and C/C, an intergranular fracture; and B, a fracture through a cobalt binder. To the left of the crack, the phenomena of grain-boundary wetting are marked. The arrows and symbol CW indicate the boundaries between tungsten carbide grains completely wetted with a cobalt binder. The arrows and symbol PW indicate the boundaries between tungsten carbide grains partially wetted with a cobalt binder. To the right of the crack, the structural elements of the “islands” of the cobalt binder are marked. Arrows and the symbol IA indicate kinks at the interface (“incoming angles”), in which the ridges of faceted tungsten carbide crystallites enter the region of the cobalt binder. The arrows and symbol OA indicate kinks at the interface (“outgoing angles”) where the cobalt binder contacts the WC/WC boundaries between tungsten carbide grains. The ellipse (or oval circle) denotes, for example, a continuous region of cobalt binder grains. For such areas, an analysis of the contour of the interphase boundary was carried out with the determination of incoming (IA) and outgoing (OA) angles. The lengths of the semiaxes of such ellipses, which characterize the dimensions of the continuous regions of the cobalt binder, were also determined. (**b**) The equilibrium shape (ECS) of a WC crystal (truncated triangular prism). Color shows different variants of ECS (**c**) Schematic cross-sectional diagram of a WC-Co polycrystal. Tungsten carbide grains are shown with green contours and white-filled. Cobalt grains are shown with blue contours and red-filled. Incoming (IA) and outgoing (OA) angles are shown. If all grains are identical hexagons in cross section, then OA = 120° and IA = 240°.

The equilibrium shape of WC crystals is the trigonal prism (see Figure 1b). In most cases, the WC grains in cemented carbides have flat facets intersecting along sharp edges forming point-like corners (see Figure 1b). 

However, in some cases, the edges and corners of WC grains become rounded such as in the WC-12 wt.% Co cemented carbides manufactured using the selective electron beam melting (SEBM) [36,37], laser powder bed fusion [38], hot isostatic pressing [39], spark plasma sintering [40], as well as by varying the carbon [41] and cobalt content [42]. The alloying of conventional cobalt binders with various metals such as Al, Ni and W can change the equilibrium shape of WC crystals. It can differ from the ideal trigonal prism and become additional facets [43]. After the alloying with ruthenium [44,45], vanadium [46], iron [22,47], rhenium [48], as well as after the addition of TaC nanoparticles [49], the WC facets also become rounded. In an extreme case, the WC crystallites completely lose their flat facets and obtain a spherical shape [50]. It can happen, for example, when the conventional cobalt-based binder is substituted with the AlSi10Mg alloy. The substitution of cobalt by the multicomponent alloys, such as in different CoCrCuFeNi, Al_0.5_CoCrCuFeNi or Al_2_CoCrCuFeNi, also lead to the strong rounding of WC grains [51].

The WC-based cemented carbides are widely used in industry and mining, as well as at the household level. Further development of cemented carbides based on tungsten carbide requires new methods for describing their microstructure, which go beyond the traditional approaches of quantitative metallography. In this work, for the first time, the outlines of the regions of the cobalt binder were chosen as a tool for describing the structure of cemented carbides. Figure 1a shows a crack that we obtained in the hard alloy WC-6 wt.% Co when the indenter is pressed. It is a good visible that the trajectory of the crack strongly depends not only on the shape of WC “capes” but also on that of the cobalt “lakes”. Therefore, we propose here a fundamentally new approach to describing the microstructure of these materials—namely, the study of the statistical geometric properties of the WC/Co interfaces in cemented carbides based on tungsten carbide (WC) and cobalt (Co) binder using machine vision elements. This is the aim of this work.

## 2. Experimental

In this work, model samples were made that imitate conventional commercial WC–Co cemented carbides with different cobalt content and different grain sizes of WC. The samples were made from a mixture of tungsten carbide and cobalt powders with an average grain size of about one micron. The powders were ball-milled in a Turbular mixer with WC-Co cemented carbide balls in hexane at a ratio of balls to powder of 6:1 with 2 wt.% paraffin for 20 h. The suspension thus obtained, after drying, was sieved to obtain the WC-Co powders. Sieving powders after drying is used to separate the powder from carbide balls employed for milling so that the sieve cells are quite coarse (nearly 1 mm). From sieved WC-Co powders, the green samples (cylinders) with a diameter of about 20 mm and a height of 5 mm were pressed. Pressed samples were sintered in a vacuum furnace at a temperature of 1400 °C for one hour. Samples for structural studies were mechanically ground on a series of silicon carbide abrasive papers and polished with diamond pastes with a decreasing grain size of 6, 3 and 1 µm, successively. The resulting sections were subjected to microscopic examination using scanning electron microscopy (SEM) and X-ray microanalysis on a Tescan Vega TS5130 MM instrument equipped with an Oxford Instruments LINK energy dispersive spectrometer. Digital images were taken in reflected electrons, where the image size was 1680 by 1680 pixels and each pixel had 256 shades of gray.

**Figure 2:**
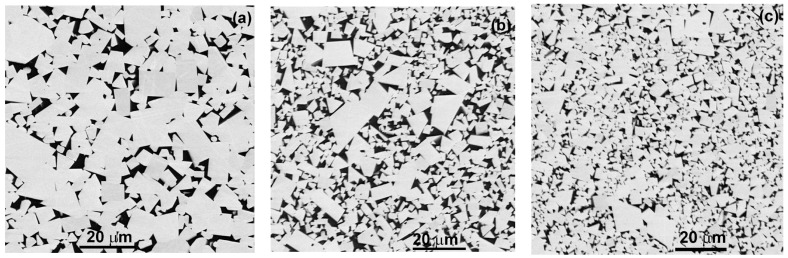
SEM micrographs of three studied samples: (**a**) WC-6 wt.% Co, (**b**) WC-11 wt.% Co and (**c**) WC-26 wt.% Co.

A series of Images In reflected electrons was obtained using SEM, in which the number of structural elements for each alloy was at least 10^5^. The structural elements are tungsten carbide and cobalt grains, boundaries between tungsten carbide grains, boundaries between cobalt grains, interphase boundaries between carbide grains tungsten and cobalt, as well as triple junctions of grain and interphase boundaries. For each of the three samples differing in tungsten carbide grain size, 100 SEM micrographs were taken. The number of photographed structural elements increased with a decrease in the WC grain size of the alloy and amounted to approximately 2 × 10^5^, 8 × 10^5^ and 4 × 10^6^, respectively, for alloys with 6, 11 and 26 wt.% Co and average WC grain sizes of 8 ± 0.2, 5 ± 0.1 and 2 ± 0.05 µm, respectively. As can be seen from the micrographs shown in Figure 2, the studied alloys contain two phases, namely, tungsten carbide and cobalt. Tungsten carbide crystallites appear white or light gray in micrographs, while the regions occupied by the cobalt bond appear dark gray or black. 

## 3. Results and Discussion

### 3.1. Image Processing

**Figure 3:**
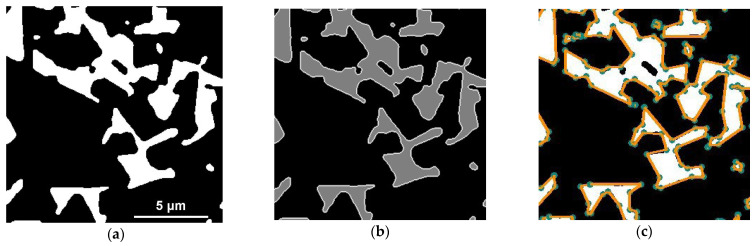
(**a**) An example of an image fragment preprocessed using the Otsu binarization algorithm. The light areas correspond to the cobalt binder; the dark areas correspond to tungsten carbide. (**b**) An example of a fully processed image. (**c**) Linearly approximated contours (highlighted in orange) of the boundaries of the cobalt binder. Green dots are breakpoints and corners of the region.

The first stage of computer analysis of images consisted in noise suppression. For this, a weighted median filter was applied [52]. The peculiarity of this algorithm is that when choosing the optimal parameters, it is possible to remove point artifacts in the image without much damage to the contours of objects. Next, it was necessary to increase the contrast at the boundary of the WC and Co regions. For this, the Otsu binarization method [53] was used. This algorithm is well suited for highlighting two different classes in an image. In our case, the first class is WC pixels, and the second class is Co pixels. Otsu’s algorithm first builds a histogram of pixel values and calculates the threshold value of the two classes so that the intra-class variance of each class is minimal. The result after applying the Otsu method is shown in Figure 3a.

The image binarized using the Otsu algorithm was passed through the Sobel filter [54], which is a discrete differential operator that calculates the approximate value of the image brightness gradient. For our task, we used the Sobel operator to obtain a gradient map that well highlights the pixel contrast transition. The boundaries of the cobalt binder in the image (Figure 3a) can be distinguished by applying the operator [55] to the image, which is one of the best image boundary detectors. Its principle of operation is based on the use of a combination of such algorithms as normal smoothing, Sobel filter [54], non-maximum suppression, threshold filtering and removal of areas not related to “strong” boundaries.

In general, image processing included the following steps: The binarized image was added to the gradient map. Then the resulting image was processed with the Kenny contour detector, and, at the output, each pixel of the image received one of three values: 0 for tungsten carbide, 127 for cobalt binder and 255 for interphase boundary tungsten carbide/cobalt (see Figure 2b). The pixels of each WC/Co boundary contour were numbered clockwise using a convolution matrix (see Figure 2) [56]. For further analysis, not the entire set of points was used but only the corner points and break points, which completely characterize the piecewise–smooth boundary. For this, the Ramer–Douglas–Peucker algorithm [57] was used. This algorithm makes it possible to reduce the number of points in a curve approximated with a large series of points. The resulting corner points were used to linearly approximate the contours of cobalt (see Figure 3c).

Figure 1a shows the elements of the microstructure, for which the above methods of numerical analysis were used for the identification. As already noted, the microstructures of the studied WC–Co alloys consist of well-faceted tungsten carbide grains (they look light in the micrograph) and a cobalt binder (which looks dark in the micrograph). Tungsten carbide has a high hardness and determines the cutting properties of hard alloys, while the cobalt binder is ductile and connects hard but brittle carbide grains. It gives the hard alloy the necessary ductility. During liquid-phase sintering, the molten cobalt binder can completely or partially wet the boundaries between tungsten carbide grains. In the case of complete wetting, the cobalt bond separates the tungsten carbide grains, and the contact angle between the cobalt bond and the WC/WC grain boundary is formally zero. Such places are shown in the micrograph with an arrow and designated as CW. In the case of partial wetting of the WC/WC boundaries, the contact angle between the cobalt binder and the WC/WC grain boundary is nonzero. Such places are shown in the micrograph by an arrow and designated as PW. On the contour of the interphase boundary between tungsten carbide and cobalt binder, kinks can be distinguished, indicated with arrows and symbols IA and OA. The kinks IA (“incoming angles”) are places where the edges of faceted tungsten carbide crystallites enter the region of the cobalt binder. The OA kinks (“outgoing angles”) are the places where the cobalt binder contacts the boundaries between the grains of the tungsten carbide. This is the case in incomplete (or partial) wetting of the WC/WC boundaries, when the contact angle is not equal to zero. Figure 1c shows a scheme of a cross-section of a two-phase polycrystal, which consists of identical regular hexagonal grains of two phases. In such an ideal case, all “outgoing angles” OA = 120° and “incoming angles” IA = 240°.

### 3.2. Angle Distribution 

The calculation of the angles at points IA and OA at the interface between the tungsten carbide and the cobalt binder was based on the scalar product. In this case, three contour points, *x*_1_, *x*_2_, *x*_3_, were selected, following one after another, and two vectors **a** = (*x*_12_–*x*_11_, *x*_22_–*x*_21_, 0) и **b** = (*x*_13_–*x*_12_, *x*_23_–*x*_23_, 0). The angle ϕ between vectors **a** and **b** was calculated with the formula *ϕ* = arccos((**a**, **b**)*/*(**a b**)). To determine whether an angle belongs to a sector less than π and more than π, we used a triple of vectors **a**, **b**, **c**, where **c** = [**a**, **b**] and the sign of the determinant *D* = [**a**, **b**]. Iteratively passing through all the contours, we obtained the values of the angles formed by the contours of the WC/Co boundaries.

**Figure 4:**
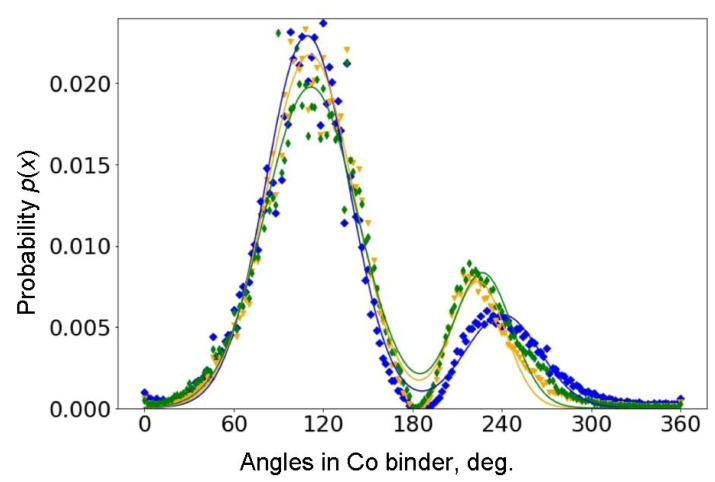
Distribution of angles on the contour of cobalt binder regions for three samples with different WC grain sizes. Blue is for small grains; orange is for medium grains; green is for coarse grains.

For each of the three samples, one hundred micrographs were processed with 2 × 10^5^, 8 × 10^5^ and 4 × 10^6^ structure elements, respectively, and a histogram of the angles at the interfacial boundary of the cobalt binder and tungsten carbide was constructed. The resulting normalized density distribution of angles is shown in Figure 4.

The symbols denote our data on the angle distribution density on the contour of the cobalt binder, and the solid curves denote the bimodal approximation of these data. Blue symbols and curves are for the sample with small grains; orange ones are for medium grains; green symbols and curves are for the coarse grains. Approximation gives the following values of average angles (see also Table 1): 112° ± 6° and 227° ± 3° for the sample with coarse grains (green color), 111° ± 2° and 224° ± 2° for medium grains (orange color), 109° ± 3° and 241° ± 2° for small grains (blue color).

The methods of computer analysis of the microstructure developed in this work made it possible for the first time to obtain curves for the distribution of angles on the contour of the interphase boundary by analyzing the boundary of the cobalt binder. The obtained curves exhibit two maxima: a high one at about 110° and a low one at about 240°. These angles, indeed, are not far from the “ideal” ones at 120° and 240° shown in Figure 1c. The high maximum corresponds to outgoing angles OA, that is, the contact angles θ between WC/WC grain boundaries (GBs) and the Co-based melt. The mean values of θ = 112°, 111° and 109° for outcoming angles are governed by the conditions of incomplete wetting of WC/WC GBs during the liquid phase sintering [58]. Only a few WC/WC GBs were completely wetted by the melt in our samples (see the small peaks close to 0° in Figure 4) [59]. The angular positions of the first maximum of θ = 112°, 111° and 109° are quite close to each other for the three studied samples of tungsten carbide with different amounts of cobalt binder and different grain sizes. This means that the conditions of GB wetting is similar in the samples with different grain sizes and different cobalt amounts. The value of θ for each GB is determined with Young’s equation σ_GB_ = cos 2 σ_IB_, where σ_GB_ is the energy of WC/WC grain boundary and σ_IB_ is the energy of the WC/Co interphase boundary. Thus, the spread of the θ values is determined mainly by the spread of GB energy σ_GB_ which, in turn, depends on the crystallographic parameters of the WC/WC grain boundaries [59]. The spread of the θ values is slightly different; this means that, most probably, the width of the energy spectrum σ_GB_ differs a little in three studied samples with different WC grain sizes. The amplitude of this maximum increases with decreasing grain size. This phenomenon can be explained by the large number of boundaries between grains of tungsten carbide with a smaller grain size. We have to underline here that the θ values measured in the sample sections are slightly different from the “true” dihedral contact angles from the Young’s equation. They are equal only in the case of complete GB wetting θ = 0°. 

The nature of the second peak of the so-called incoming angles IA around 240° is different. It characterizes the edges of the WC “capes” contacting the cobalt “lakes”. These are the angles between the faces of the individual WC crystallites in contact with the Co binder [60]. The values of these angles are determined by the shape of WC grains (shown in Figure 1b) and are not associated with grain boundary phenomena in the structure. In contrast to the first peak, the angular position of the second peak differs markedly for three different samples. The difference in the angular positions of the second peak can also be due to the possible influence of Co content on the shape of WC crystals [45,46,47,48,49,50,51,52,53,54]. This feature can also be explained by the fact [60] that, in the samples, the height of WC crystals (see Figure 1) prevails over their width. In contrast to the left peak, the amplitude of the right peak increases with the WC grain size. This can be explained by the fact that, at small grain sizes, there is less space between adjacent WC/WC boundaries for the edges of individual WC crystallites to directly contact the cobalt binder. This conclusion is indirectly confirmed with the following analysis of the geometry of the cobalt regions.

### 3.3. The Length Distribution for Ellipse Semiaxes

The standard method for studying the dimensions of structural elements is the method of selecting the maximum segments and estimating the area occupied by the cobalt binder and the effective radius of the cobalt binder region associated with it [61]. Computer vision allows one to determine more informative numerical estimates. As a first example, we applied the next order of magnitude approximation to the size and shape of the cobalt regions, namely their approximation with elliptic curves.

**Figure 5:**
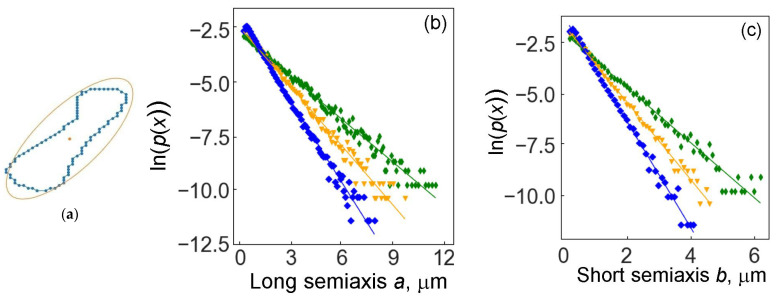
(**a**) Scheme of an ellipse around the points of the contour. The distribution of the lengths of the semiaxes of such ellipses for the cobalt binder for three samples with different WC grain sizes: (**b**) long semiaxis, (**c**) short semiaxis. The vertical axis shows the logarithms of the distribution probability. Blue is for small WC grains; orange is for medium WC grains; green is for coarse WC grains.

To determine the width and length of the cobalt binder region, we describe an ellipse around the contour points (see the ellipse in the micrograph in Figure 1 and the diagram in Figure 5a) using the Khachiyan algorithm [62]. Passing through all the previously selected contours of cobalt islands, we obtain the distributions of the lengths of the semiaxes of the desired ellipses. Figure 5 shows the distribution densities of the major and minor semiaxes for three samples, which are well approximated by the exponential distribution. The parameters of the exponential distribution of semiaxes are shown in Table 2.

The probability *p*(*x*) of semiaxis lengths for all samples decreases exponentially with increasing semiaxis length. The length of the semiaxes decreases at different rates in samples with different grain sizes of tungsten carbide. The semiaxis length decreases most rapidly in specimens with coarse WC grains. This fact is already rather nontrivial, since we are talking about the sizes of the regions of the cobalt binder and not the grains of tungsten carbide. At the same time, the volume fraction of the cobalt binder is the highest in the sample with fine WC grains.

The second nontrivial and unexpected fact is that the lines *p*(*x*) in logarithmic coordinates for three samples with different grain sizes intersect at one point at a short semiaxis length of about 0.7 µm and at a long semiaxis length of about 1.1 µm. This fact outwardly resembles, for example, the intersection at one point of the temperature dependences of the coefficients of grain boundary diffusion in Arrhenius coordinates for grain boundaries with different misorientation angles.

The identification of the characteristic grain size is a typical task in materials science, for example, the Saltykov method [61] for obtaining characteristic segments. In our approach, we obtained two characteristic lengths for cobalt binder islands, the major and minor semiaxes of the circumscribed ellipse.

**Figure 6:**
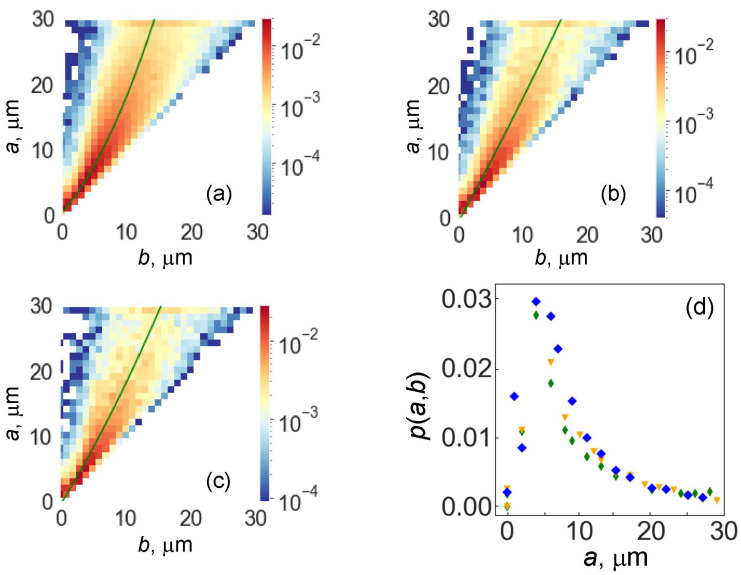
Two-dimensional distribution densities *P*(*a*,*b*) of the lengths of the semiaxes *a* and *b* of cobalt islands: (**a**) small WC grains, (**b**) medium WC grains, (**c**) coarse WC grains. The color scale on the right represents the values of *P*(*a*,*b*). The solid lines are for the distribution ridge. (**d**) The value of *P*(*a*,*b*) on the distribution ridge as a function of the major semiaxis: blue is for small grains, orange is for medium grains, green is for coarse grains.

The obtained statistical data make it possible to expand the approach and obtain a joint distribution of the lengths of the semiaxes of ellipses approximating the shape of the islands of cobalt binder. The respective distributions are shown in Figure 6.

The color corresponds to the probability density of the joint values of the major and minor semiaxes. The upper-left corner corresponds to complete wetting. The maximum corresponds to almost rounded regions of the cobalt binder. 

We also highlighted the “ridge” of this distribution, which characterizes the correlations between the semiaxes (shown in Figure 6a–c with a solid line, a second-order polynomial). Figure 6d shows the relative density of the two-dimensional distribution on the “ridges” of the two-dimensional distributions. A color is plotted along the vertical, which reflects the distribution density of almost rounded regions (more precisely, cobalt regions with almost equal axes). It is interesting that these distribution densities practically coincide. This means that, within the accuracy of the estimates made (the step along the semiaxes is 1 μm), the relative frequency of almost rounded regions is the same for three different samples, both in terms of the size of the regions and the relative mass percentage of the cobalt binder. 

In Table 3, the bulk properties of the studied WC-Co alloys are given with different cobalt content and different grain size. The density of the alloys obviously decreases with increasing cobalt concentration because of a difference in density between W (19,300 kg/m^3^) and Co (8900 kg/m^3^). Vickers hardness does not change much. In the cemented carbides with various compositions, it can scatter between 1000 and 2200 HV [63]. However, the coercive force is the most sensitive to the structural features (since cobalt has ferromagnetic properties) [64]. It is known that the coercive force of WC–Co cemented carbides is controlled by the movement of domain walls during demagnetization and, thus, by the morphology of WC/Co interphases [64]. It can be concluded, therefore, that, in our case, the coercive force strongly increases (Table 3) with the decreasing lengths of the major and minor semiaxes of cobalt islands (Table 2).

Figure 1a shows a crack that we obtained in the hard alloy WC-6 wt.% Co when the indenter is pressed in. It is clearly seen that this crack has a rather complex shape. Partially, it passes between the grains of tungsten carbide (C/C in Figure 1a); in these places, the crack is usually flat. This is the so-called intergranular fracture. In other places, the crack cuts, splitting the grains of tungsten carbide (C in Figure 1a). This is the so-called transcrystalline fracture. The crack can also pass along the interface separating the tungsten carbide grains from the cobalt binder (C/B in Figure 1a). Finally, the crack can go through the volume of the cobalt binder (B in Figure 1a).

Deviation of a crack from a flat trajectory is the main mechanism that determines the crack resistance of WC-based cemented carbides [2]. The strong bending of the crack on WC grains is determined by the characteristics of the crystal. The WC crystal belongs to a close-packed hexagonal structure and has only one {1010} 〈1123〉 slip family in the unit cell and only four independent slip systems in this slip family (Figure 1b). Therefore, when the crack propagates towards the WC grains, the intercrystalline fracture prevails over the transgranular one. The point is that it is almost unbelievable that the plane and direction of sliding will be the same in adjacent WC grains. In turn, crack propagation in the spaces between WC crystallites will strongly depend on their shape. In addition, crack trajectories are more tortuous near larger WC grains, and cracks are more likely to bend and branch. The thickness of cobalt interlayers in coarse-grained alloys is higher than in fine-grained alloys, which leads to a higher probability of preventing crack propagation if their front hits a thick cobalt interlayer (Figure 1a). Moreover, the shape of the areas of the cobalt binder should influence the crack trajectory. Thus, the fundamentally new methods developed here for describing the shape and mutual arrangement of tungsten carbide grains, as well as the cobalt binder surrounding them, will make it possible to significantly advance efforts when using them in improving the properties and developing new materials based on tungsten carbide. 

## 4. Conclusions

In this paper, we used the machine vision methods to obtain informative statistical data on the topology of the interfaces between tungsten carbide grains (WC) and cobalt matrix (Co) in WC–Co cemented carbides. For the first time, the shape of the “lakes” of the cobalt binder was chosen as a tool for describing the structure of cemented carbides. Numerical processing of micrographs of cross sections of three WC–Co alloys, which differ in the average size of WC grains and the amount of cobalt, was carried out. The distribution density of the angles in the contours of cobalt “lakes” is bimodal. The peaks close to θ = 110° (so-called outcoming angles) correspond to the contacts between the cobalt binder and the WC/WC grain boundaries. The value close to the 110° is determined by the incomplete wetting of WC/WC grain boundaries by the cobalt-rich melt during liquid-phase sintering. The amount of completely wetted WC/WC grain boundaries (contact angles close to θ = 0°) is very small. 

The peaks close to θ = 240° (or incoming angles) correspond to the WC “capes” contacting the cobalt “lakes” and are determined by the angles between facets of WC crystallites. The distribution density of the linear dimensions of the regions of the cobalt binder, approximated with ellipses, was obtained. The distribution density exponentially decreases with the lengths of the semi-axes of the ellipsoid, approximating the area of the cobalt binder. The probability *p*(*x*) of semiaxis lengths of ellipses for all three samples decreases exponentially with an increasing semiaxis length. The length of the semiaxes decreases at different rates in samples with different sizes of WC grains. The semiaxis length decreases most rapidly in specimens with coarse WC grains. This fact is rather nontrivial, since we are talking about the sizes of the regions of the cobalt binder and not the WC grains. The spread of the θ value around 240° may indicate the change of the shape of the WC equilibrium shape with the cobalt amount. The possible connection between the obtained data on the shape of cobalt areas and the crack trajectories in cemented carbides is discussed.

## Figures and Tables

**Table 1:** The position of the distribution peaks in Figure 4.

WC Grain Size in the Sample	Left Peak	Right Peak
Coarse WC grains, 8 ± 0.2 µm, green color	112° ± 6°	227° ± 3°
Medium WC grains, 5 ± 0.1 µm, orange color	111° ± 2°	224° ± 2°
Fine WC grains, 2 ± 0.05 µm, blue color	109° ± 3°	241° ± 2°

**Table 2:** Characteristics of cobalt regions in alloys with different WC grain sizes (see Figure 5).

WC Grain Size and Cobalt Content	Number of Studied Areas	Long Semiaxis, µm	Short Semiaxis, µm
Coarse grains, 6 wt.% Co, green color	17026	1.48	0.75
Medium grains, 11 wt.% Co, orange color	31243	1.07	0.57
Fine grains, 26 wt.% Co, blue color	87934	0.84	0.47

**Table 3:** Properties of the studied WC-Co alloys.

Co Content, wt.%	WC Grain Size, µm	Density, kg/m^3^	Vickers Hardness, HV	Coercive Force, Oe
6	8 ± 0.2	14,900 ± 5	1090 ± 2	70 ± 0.3
11	5 ± 0.1	14,520 ± 5	1030 ± 2	62 ± 0.3
26	2 ± 0.05	13,480 ± 5	1060 ± 2	91 ± 0.3

## Data Availability

Data are contained within the article.

## References

[1] Schröter K. (1923). DRP 420.689: Sintered Hardmetal Alloy And Procedure For Its fabrication. https://patents.google.com/patent/DE420689C/en.

[2] Cardarelli F. (2008). Materials Handbook: A Concise Desktop Reference.

[3] Haynes W.M. (2014). CRC Handbook of Chemistry and Physics.

[4] Swab J.J., Wright J.C. (2016). Application of ASTM C1421 to WC-Co fracture toughness measurement. Int. J. Refract. Met. Hard Mater..

[5] Konyashin I., Straumal B.B., Ries B., Bulatov M.F., Kolesnikova K.I. (2017). Contact angles of WC/WC grain boundaries with binder in cemented carbides with various carbon content. Mater. Lett..

[6] Konyashin I., Ries B., Hlawatschek D., Zhuk Y., Mazilkin A., Straumal B., Dorn F., Park D. (2015). Wear-resistance and hardness: Are they directly related for nanostructured hard materials?. Int. J. Refract. Met. Hard Mater..

[7] Sahoo B.N., Mohanty A., Gangopadhyay S., Vipindas K. (2020). An insight into microstructure and machining performance of deep cryogenically treated cemented carbide inserts. J. Manuf. Process..

[8] Gille G., Bredthauer J., Gries B., Mende B., Heinrich W. (2000). Advanced and new grades of WC and binder powder—Their properties and application. Int. J. Refract. Met. Hard Mater..

[9] Chang S.H., Chang M.H., Huang K.T. (2015). Study on the sintered characteristics and properties of nanostructured WC-15 wt.% (Fe-Ni-Co) and WC-15 wt.% Co hard metal alloys. J. Alloys Compd..

[10] Bounhoure V., Lay S., Coindeau S., Norgren S., Pauty E., Missiaen J.M. (2015). Effect of Cr addition on solid state sintering of WC-Co alloys. Int. J. Refract. Met. Hard Mater..

[11] Huang Z., Ren X.R., Liu M.X., Xu C., Zhang X.H., Guo S.D., Chen H. (2017). Effect of Cu on the microstructures and properties of WC-6Co cemented carbides fabricated by SPS. Int. J. Refract. Met. Hard Mater..

[12] Lin N., Jiang Y., Zhang D.F., Wu C.H., He Y.H., Xiao D.H. (2011). Effect of Cu, Ni on the property and microstructure of ultrafine WC-10Co alloys by sinter-hipping. Int. J. Refract. Met. Hard Mater..

[13] Puga J.B., Fernandes C.M., Vieira M.T., Senos A.M.R. (2013). Morphological characterization by scanning electron microscopy of WC powder particles coated with Cu. Microsc. Microanal..

[14] Chen C.S., Yang C.C., Chai H.Y., Yeh J.W., Chau J.L.H. (2014). Novel cermet material of WC/multi-element alloy. Int. J. Refract. Met. Hard Mater..

[15] Shon I.J. (2016). Effect of Al on sintering and mechanical properties of WC-Al composites. Ceram. Int..

[16] Wittmann B., Schubert W.D., Lux B. (2002). WC grain growth and grain growth inhibition in nickel and iron binder hardmetals. Int. J. Refract. Met. Hard Mater..

[17] Norgren S., Garcia J., Blomqvist A., Yin L. (2015). Trends in the P/M hard metal industry. Int. J. Refract. Met. Hard Mater..

[18] Ahmadian M., Wexler D., Calka A., Chandra T. (2003). Liquid phase sintering of WC-FeAl and WC-Ni_3_Al composites with and without boron. Mater. Sci. Forum.

[19] Fernandes C.M., Senos A.M.R., Vieira M.T., Antunes J.M. (2008). Mechanical characterization of composites prepared from WC powders coated with Ni rich binders. Int. J. Refract. Met. Hard Mater..

[20] Fernandes C.M., Senos A.M.R. (2011). Cemented carbide phase diagrams: A review. Int. J. Refract. Met. Hard Mater..

[21] Gonzalez R., Echeberria J., Sanchez J.M., Castro F. (1995). WC-(Fe,Ni,C) hardmetals with improved toughness through isothermal heat-treatments. J. Mater. Sci..

[22] Schubert W.D., Fugger M., Wittmann B., Useldinger R. (2015). Aspects of sintering of cemented carbides with Fe-based binders. Int. J. Refract. Met. Hard Mater..

[23] Garcia J. (2011). Investigations on kinetics of formation of fcc-free surface layers on cemented carbides with Fe-Ni-Co binders. Int. J. Refract. Met. Hard Mater..

[24] Habibi Rad M., Ahmadian M., Golozar M.A. (2012). Investigation of the corrosion behavior of WC–FeAl–B composites in aqueous media. Int. J. Refract. Met. Hard Mater..

[25] Fernandes C.M., Senos A.M.R., Vieira M.T. (2012). Versatility of the sputtering technique in the processing of WC-Fe-Ni-Cr composites. Surf. Coat. Tech..

[26] Zhao Z.Y., Liu J.W., Tang H.G., Ma X.F., Zhao W. (2015). Investigation on the mechanical properties of WC-Fe-Cu hard alloys. J. Alloys Compd..

[27] Razavi M., Rahimipour M.R., Yazdani-Rad R. (2011). Synthesis of Fe-WC nanocomposite from industrial ferrotungsten via mechanical alloying method. Adv. Appl. Ceram..

[28] Shon I.J. (2016). Rapid consolidation of nanostructured WC-FeAl hard composites by high-frequency induction heating and its mechanical properties. Int. J. Refract. Met. Hard Mater..

[29] Hanyaloglu C., Aksakal B., Bolton J.D. (2001). Production and indentation analysis of WC/Fe-Mn as an alternative to cobalt-bonded hardmetals. Mater. Charact..

[30] Maccio M.R., Berns H. (2012). Sintered hardmetals with iron-manganese binder. Powder Metall..

[31] Pittari III J.J., Murdoch H.A., Kilczewski S.M., Hornbuckle B.C., Swab J.J., Darling K.A., Wright J.C. (2018). Sintering of tungsten carbide cermets with an iron-based ternary alloy binder: Processing and thermodynamic considerations. Int. J. Refract. Met. Hard Mater..

[32] Machado I.F., Girardini L., Lonardelli I., Molinari A. (2009). The study of ternary carbides formation during SPS consolidation process in the WC-Co-steel system. Int. J. Refract. Met. Hard Mater..

[33] Oliveira A.B., Bastos A.C., Fernandes C.M., Pinho C.M.S., Senos A.M.R., Soares E., Sacramento J., Zheludkevich M.L., Ferreira M.G.S. (2015). Corrosion behaviour of WC-10% AISI 304 cemented carbides. Corros. Sci..

[34] Fernandes C.M., Senos A.M.R., Vieira M.T. (2003). Sintering of tungsten carbide particles sputter-deposited with stainless steel. Int. J. Refract. Met. Hard Mater..

[35] Fernandes C.M., Vilhena L.M., Pinho C.M.S., Oliveira F.J., Soares E., Sacramento J., Senos A.M.R. (2014). Mechanical characterization of WC-10 wt.% AISI 304 cemented carbides. Mater. Sci. Eng. A.

[36] Wang J., Han Y., Zhao Y., Li X., Yi D., Guo Z., Cao Y., Liu B., Tang H.P. (2022). Microstructure and properties of WC-12Co cemented carbide fabricated via selective electron beam melting. Int. J. Refr. Met. Hard Mater..

[37] Akerman J., Ericson T. (1997). Cemented Carbide Body with Improved High Temperatures and Thermomechanical Properties. U.S. Patent.

[38] Zhang L., Hu C., Yang Y., Misra R.D.K., Kondoh K., Lu Y. (2022). Laser powder bed fusion of cemented carbides by developing a new type of Co coated WC composite powder. Add. Manufact..

[39] Rabouhi H., Eyidi D., Khelfaoui Y., Khireddine A. (2022). Microstructural and mechanical characterisation of WC–Co alloys elaborated by liquid phase sintering and hot isostatic pressing: Study of WC crystallites size evolution. Can. Metal. Quart..

[40] Chen Z., Wang Z., Wang B., Yuan J., Huang L., Yin Z. (2021). Microstructure and properties of WC-8Co cemented carbides prepared by multiple spark plasma sintering. Int. J. Appl. Ceram. Technol..

[41] Fries S., Burkamp K., Broeckmann C., Richter S., Westermann H., Süess B. (2022). Influence of carbon content on fatigue strength of cemented carbides. Int. J. Refr. Met. Hard Mater..

[42] Shi K.-H., Zhou K.-C., Li Z.-Y., Zan X.-Q., Dong K.-L., Jiang Q. (2022). Microstructure and properties of ultrafine WC–Co–VC cemented carbides with different Co contents. Rare Met..

[43] Edtmaier C., Wolf M., de Oro Calderon R., Schubert W.-D. (2021). Effect of nickel on the formation of γ/γ′ microstructures in WC/Co–Ni–Al–W. Int. J. Refr. Met. Hard Mater..

[44] Zeng H., Liu W., Han F., Wie C. (2022). Microstructures and properties of WC–10Co tuned by Ru integration and ball-milling. Mater. Sci. Technol..

[45] Zeng H., Liu W., Wei C. (2022). Influence of Ru on the microstructure and performance of WC–Co cemented carbides. Mater. Sci. Technol..

[46] Ye X., Xiang C., Nie H., Lei H., Du Y., Xing W., Luo J., Yu Z. (2022). Facet-dependent interfacial segregation behavior of V-doped WC-Co cemented carbides. Ceram. Intern..

[47] Agte C. (1957). Entwicklung der Hartmetalltechnik waehrend der letzten Jahre in der Deutschen Demokratischen Republik. Neue Huette.

[48] Jing K., Guo Z., Hua T., Xiong J., Liao J., Liang L., Yang S., Yi J., Zhang H. (2022). Strengthening mechanism of cemented carbide containing Re. Mater. Sci. Eng. A.

[49] Hu H., Liu X., Chen J., Lu H., Liu C., Wang H., Luan J., Jiao Z., Liu Y.X. (2022). Song, X. High-temperature mechanical behavior of ultra-coarse cemented carbide with grain strengthening. J. Mater. Sci. Technol..

[50] Hu Z., Zhao Z., Deng X., Lu Z., Liu J., Qu Z., Jin F. (2022). Microstructure and mechanical behavior of cemented carbide with Al alloy binder fabricated by selective laser melting. Int. J. Refr. Met. Hard Mater..

[51] Mueller-Grunz A., Alveen P., Rassbach S., Useldinger R., Moseley S. (2019). The manufacture and characterization of WC-(Al)CoCrCuFeNi cemented carbides with nominally high entropy alloy binders. Int. J. Refr. Met. Hard Mater..

[52] Brownrigg D.R.K. (1984). The weighted median filter. Commun. ACM.

[53] Nobuyuki O. (1979). A threshold selection method from gray-level histograms. IEEE Trans. Syst. Man Cyber..

[54] Farid H., Simoncell E.P., Sommer G., Daniilidis K., Pauli J. (1997). Optimally rotation-equivariant directional derivative kernels. Proceedings 7th International Conference on Computer Analysis of Images and Patterns.

[55] Rong W., Li Z., Zhang W., Sun L. (2014). An improved CANNY edge detection algorithm. Proc. IEEE Int. Conf. Mechatron. Automat..

[56] Suzuki S., Abe K. (1985). Topological structural analysis of digitized binary images by border following. Comp. Vis. Graph. Image Proc..

[57] John H., Jack S. Speeding up the Douglas-Peucker Line-Simplification Algorithm. (CiteSeerX, Vancouver, 1992). https://tildesites.bowdoin.edu/~ltoma/teaching/cs350/spring04/Handouts/hershberger92speeding.pdf.

[58] Rabkin E.I., Shvindlerman L.S., Straumal B.B. (1991). Grain boundaries: Phase transitions and critical phenomena. Int. J. Mod. Phys. B.

[59] Noskovich O.I., Rabkin E.I., Semenov V.N., Straumal B.B., Shvindlerman L.S. (1991). Wetting and premelting phase transitions in 38°[100] tilt grain boundaries in (Fe–12at.%Si) Zn alloy in the vicinity of the A2–B2 bulk ordering in Fe–12at.%Si alloy. Acta Met. Mater..

[60] Konstantinova E.P., Shchur L.N. (2023). Algorithm for density of angle distribution in random sections of polyhedron. Powder Metall..

[61] Saltykov S.A. (1976). Stereometric Metallography.

[62] Götschel M., Lovász L., Schrijver A. (1993). The ellipsoid method. Geometric Algorithms and Combinatorial Optimization. Algorithms and Combinatorics.

[63] Straumal B., Konyashin I. (2023). WC-Based cemented carbides with high entropy alloyed binders: A review. Metals.

[64] Sundin S., Haglund S. (2000). A comparison between magnetic properties and grain size for WC/Co hard materials containing additives of Cr and V. Int. J. Refr. Met. Hard Mater..

